# Medicinal Plants Used in the Treatment of Mental and Neurological Disorders in Ghana

**DOI:** 10.1155/2018/8590381

**Published:** 2018-12-20

**Authors:** Patrick Amoateng, Emmanuel Quansah, Thomas K. Karikari, Alex Asase, Dorcas Osei-Safo, Kennedy Kwami Edem Kukuia, Isaac Kingsley Amponsah, Alexander K. Nyarko

**Affiliations:** ^1^Department of Pharmacology & Toxicology, School of Pharmacy, College of Health Sciences, University of Ghana, P.O. Box LG 43, Legon, Accra, Ghana; ^2^Pharmacology, School of Health and Life Sciences, De Montfort University, Leicester LE1 9BH, UK; ^3^School of Life Sciences, University of Warwick, Coventry CV4 7AL, UK; ^4^Midlands Integrative Biosciences Training Partnership, University of Warwick, Coventry CV4 7AL, UK; ^5^Department of Plant and Environmental Biology, School of Biological Sciences, College of Basic & Applied Sciences, University of Ghana, P.O. Box LG 55, Legon, Accra, Ghana; ^6^Department of Chemistry, School of Physical and Mathematical Sciences, College of Basic and Applied Sciences, University of Ghana, P.O. Box LG 56, Legon, Accra, Ghana; ^7^Department of Pharmacognosy, Faculty of Pharmacy & Pharmaceutical Sciences, College of Health Sciences, Kwame Nkrumah University of Science & Technology, Kumasi, Ghana

## Abstract

**Ethnopharmacological Relevance:**

Mental and neurological disorders are a serious public health challenge globally, particularly in developing countries where cultural factors and limited access to standard healthcare have led to a reliance on traditional medicines. However, ethnopharmacological characterization of traditional medicines used to treat these diseases is lacking. In this study, an ethnobotanical description of plant species used in treating mental and neurological disorders in Ghana and an update of their experimentally validated pharmacological relevance are provided.

**Materials and Methods:**

Two hundred herbalists agreed to participate but sixty-six specialized in treating mental and neurological disorders were interviewed on their traditional medical practice. Literature review was conducted to verify the experimentally validated pharmacological importance of the reported plants.

**Results:**

Thirty-two plant species belonging to twenty-eight families were identified. Most plant species had either analgesic (50%), anxiolytic (18.8%), or anticonvulsant (15.6%) properties. Others had reported sedative, anti-Alzheimer's disease, motor coordination, antipsychotic, antidepressant, cognitive enhancement, and neuroprotective properties. While* Ageratum conyzoides *L. (Asteraceae) and* Ocimum gratissimum *L. (Lamiaceae) were the most commonly mentioned species with analgesic properties,* Lantana camara *L. (Verbenaceae) was the most-reported anxiolytic product, with* Cymbopogon citratus *DC. (Gramineae)*, Mangifera indica *L.,* Tetrapleura tetraptera *Schum Taub. (Fabaceae), and* Persea Americana *Mill (Lauraceae) being the most studied anticonvulsants.

**Conclusions:**

This study provides the first report specifically on medicinal plants used in treating mental and neurological disorders in Ghana. Most of the identified plants have been scientifically confirmed to possess neuro- and psychopharmacological properties and may serve as templates for drug development.

## 1. Introduction

The World Health Organization (WHO) estimates that more than one billion people suffer from central and peripheral nervous system (CNS/PNS) disorders globally [1, 2]. These diseases include Parkinson's disease, epilepsy, schizophrenia, bipolar disorder, Alzheimer's disease and other dementias, neuroinfections, brain tumors, traumatic disorders, and cerebrovascular diseases such as stroke and migraine. More than 6 million people reportedly die each year due to stroke, with over 80% of these deaths occurring in low- and middle-income countries [2]. Moreover, although little research attention has been paid to diseases such as schizophrenia, bipolar disorder, and other psychotic disorders in Africa, some studies have shown that schizophrenia is a major psychiatric diagnosis leading to in-patient admissions on these continents [3–6]. In addition, the CNS/PNS disease burden in Africa is exacerbated by the numerous but understudied neurological impairments associated with common tropical diseases such as the neglected tropical diseases [7].

Ghana is host to a wide array of medicinal flora and takes pride in the longstanding cultural use of traditional and alternative medicines (TAMs), as exhibited by the several published works on the ethnobotanical use of TAMs in the country [8–11]. Nonetheless, there are concerns about the safety and efficacy claims of some TAMs [12]. In order to address these concerns while enhancing the therapeutic potentials of TAMs and ensuring minimum adverse effects, the Ghanaian Government, academics, and TAM practitioners have institutionalized measures to regulate herbal medicine practice and also integrate TAMs into the mainstream healthcare system. For example, the Ghana Federation of Traditional Medical Practitioners Association (GHAFTRAM) was established in 1999 to help modernize, restructure, and regulate the traditional medical industry in the country [13]. GHAFTRAM has members from all parts of Ghana, working together towards advancing the development of TAMs. In addition, an undergraduate program in herbal medicine which complements university training with hands-on internships at a herbal medicine research centre as well as herbal and allopathic practitioners has been established [14]. On passing their professional qualifying examination, graduates are certified and regulated by Ghana's Traditional Medical Practice Council, and some are employed by the Government to practice as medical herbalists in herbal clinics established within public hospitals to work in partnership with medical and allied health staff to provide curative and preventive medical care [12, 14].

The foregoing measures emphasize that TAMs continue to play a significant role in the treatment of various disorders including those of CNS/PNS origin [15–17]. However, there have been no studies focusing primarily on the documentation of traditional methods of treating mental and neurological disorders in Ghana, and how these may inform healthcare practice, policy, and drug development. Consequently, comprehensive information on plant species, plant parts used, cultural practices, and methods of preparation and use of these TAMs are lacking. Moreover, the therapeutic potential, CNS properties, and the safety profile of most of these products are largely unknown. The present study sought to address this knowledge gap by using a guided survey to document TAMs used in the treatment of mental and neurological disorders in Ghana based on traditional knowledge. Moreover, we aimed to ascertain the scientifically confirmed pharmacological relevance of these medicinal products that may justify their clinical use and further research to isolate compounds of interest to drug discovery and development. Specifically, the study was aimed at (a) identifying commonly used TAMs for CNS/PNS disorders and their modes of preparation and (b) documenting the therapeutic potentials of these products.

## 2. Materials and Methods

### 2.1. Selection of Participants, Obtaining prior Informed Consent, Ethical Approval, and Data Collection

An ethnobotanical approach was used to explore the knowledge and treatment practices of mental and nervous system disorders by traditional medical practitioners (TMPs) from various districts and subdistricts of the Greater Accra and Brong-Ahafo regions of Ghana. Study participants were TMPs who were all members of GHAFTRAM attending a meeting in Accra. The study objectives, method, and planned use of information were explained to the TMPs before the interviews. Among the 200 TMPs present at the GHAFTRAM meeting, 66 were included in this study. The excluded delegates were not specialized in the treatment of mental and neurological diseases, as they found such patients quite difficult to manage.

A guided questionnaire interview approach was used: during the interviews conducted in both English and Twi, a local Ghanaian dialect, information on the types and parts of plant materials used, the methods of preparation, the local names of plants, and the mode of administration of herbal products were obtained. To be included in the interview, one had to be a (a) TMP practicing in Ghana, potentially treating mental and neurological diseases directly or having some level of knowledge on products used in treating such patients or (b) registered member of GHAFTRAM willing to participate in the survey. Approval for this study was granted by the Scientific and Technical Committee of the Noguchi Memorial Institute for Medical Research, Accra, Ghana, reference number STC-4 (2) 2013-14. Prior to the study, permission was granted from the leadership of GHAFTRAM and all participants duly signed informed consents.

### 2.2. Data Management and Analysis

A list of the plants obtained from the survey was subjected to thorough review using Internet search engines (such as google scholar) and journal databases such as Medline, Embase, Scopus, and Pubmed to confirm their therapeutic potential. A search of Ghanaian and West African herbal pharmacopoeias was done using the following search terms: “*neurological disorders*”, “*psychiatric disorders*”, “*schizophrenia*”, “*Parkinson's disease*”, “*Alzheimer's disease*”, and “*mental disorders*” in combination with either “*Ghana*”, “*West Africa*”, or “*Africa*”.

Data obtained from the ethnobotanical study were analyzed using the Statistical Package for Social Sciences version 22.0 for Windows.

## 3. Results

### 3.1. Sociodemographic Characteristics of Respondents

In total, 66 TMPs were interviewed: 65 and 1 from the Greater Accra and Brong-Ahafo regions, respectively. About 56.1% were males and 43.9% were females. About 40.9% of the TMPs were either 50-59-year-old or 60 years and above (27.3%). In addition, while 65.2% were married, 22.7% were single, 10.6% were widowed, and 1.5% were divorced. Most TMPs had either primary school (53.1%) or secondary school (28.1%) education (Table 1); only 10.9% had some form of postsecondary education.

### 3.2. Source of the Knowledge of Herbal Medicine Practice and Duration of Practice

**Figure 1:**
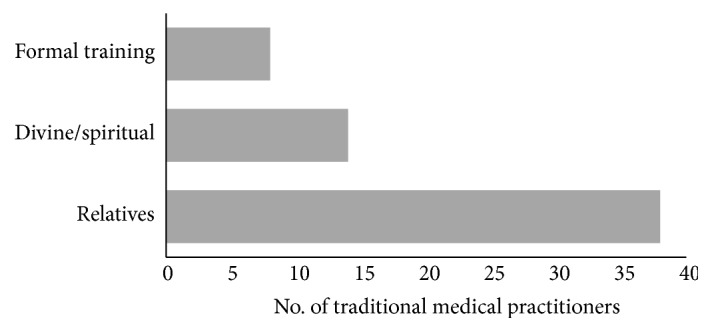
Source of traditional medical practitioners' knowledge for the treatment of mental and nervous system disorders in Ghana.

The TMPs' knowledge of traditional healing, including knowledge to treat mental and neurological disorders, was mainly acquired from relatives (Figure 1). About 36.5% had practiced for 16-20 years, while 27% had practiced for 1-5 years (Table 2).

### 3.3. Treatment of Mental and Neurological Diseases

Most of the TMPs (60.6%) had specific herbs for treating a variety of mental and neurological disorders. However, only 36.4% had actually treated such patients. Out of these, 19.7% had treated a maximum of 5 patients, with only 1.5% having treated more than 20 patients. Overall, 31.8% of the treated patients had completely recovered (Table 3).

### 3.4. Species, Medicinal Uses, and Experimentally Validated Pharmacological Relevance of Plants

**Figure 2:**
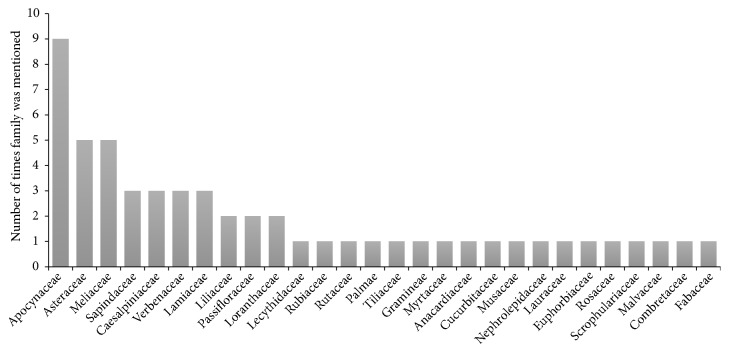
Plant families commonly used in the treatment of mental and nervous system disorders in Ghana.

**Figure 3:**
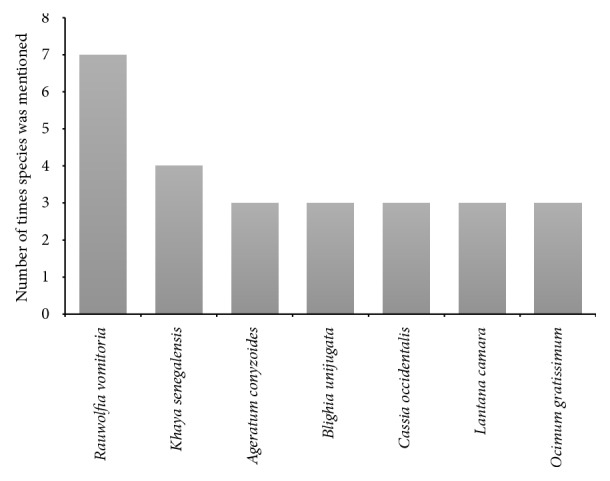
Most frequently used plant species in the treatment of mental and nervous system disorders in Ghana (only species with three or more mentions are shown).

In all, 32 plant species were provided by the TMPs and these came from 28 different plant families (Table 4). The families Apocynaceae, Asteraceae, and Meliaceae were the most mentioned plant families, with Apocynaceae having the highest frequency of mentions and Asteraceae having the highest number of plant species (Figure 2).* Rauwolfia vomitoria* Afz. was the plant with the highest frequency of mention (mentioned 7 times; Figure 3). About 66% of the plants (21 species) used have been previously reported to have neuropharmacological activities. Half of the identified TAMs had analgesic (50%) properties, with the others having anxiolytic (18.8%), sedative (6.3%), anticonvulsant (15.6%), and antidepressant (9.4%) properties.* R. vomitoria* Afz., belonging to the family* Apocynaceae,* however, has been previously reported to have antipsychotic properties [16, 18] (Table 5).

### 3.5. Preparation and Administration of Herbal Products

The TAMs were prepared mostly as mixtures of two or more species. In some cases, however, the products were administered as monopreparations (prepared using a single plant species). The mode of preparation employed included decoction, infusion, and maceration, with decoction being the commonest (Table 4). While roots, fruits, flowers, stems, stem barks, whole plant of shrubs, etc. were all used in the preparation of these products (all together 42.4%), leaves (57.6%) were the commonest plant part used. The Ghanaian vernacular names of the plant species are listed in Table 5.

Given that most TMPs do not preserve these TAMs, they generally prepared the products only when required. The products were administered orally, nasally, or applied on the forehead for periods ranging from one week to several years or until the patient recovers. The TMPs mostly used patient feedback and disappearance of symptoms to assess treatment outcomes. Where there is only a partial recovery or treatment failure, the patients are often referred to the nearest hospital.

## 4. Discussion

Mental and neurological disorders remain a major public health concern [2]. The disease burden is even more prominent in the developing world, including Ghana [3, 5, 6]. Recent discoveries and clinical usage of the anticancer agent taxol and the antimalarial artemisinin derived from plants have boosted interest in natural products as templates for the development of novel drug scaffolds [19, 20]. TAMs are widely accepted in African communities and there appears to be an increasing reliance on these products [13]. In Ghana, TAMs are used as the main treatment paradigm for a variety of diseases, but they are also used as complements to other medicines or as dietary supplements [21]. However, thorough examination and documentation of the medicinal properties of these products against mental and neurological disorders is lacking.

In the present study, several plant species (32 species) used by local TMPs to treat mental and neurological disorders were reported, with most species belonging to the families Asteraceae, Apocynaceae, and Meliaceae. These are large and widespread plant families with several species. In particular, the Asteraceae family is of great importance due to its high numbers of medicinal species used in the treatment of a wide array of diseases including tuberculosis, malaria, and inflammatory disorders [11, 15, 22]. Members of the Asteraceae family are also known for their wide range of economically important products including cooking oils and phytochemicals such as sesquiterpene lactones, alkaloids, and tannins [23]. The family Apocynaceae also has a wide range of species that are of pharmacological importance, with some members synthesizing alkaloids useful against high blood pressure and inflammation and others synthesizing cardiac glycosides that affect heart function [24]. The family Meliaceae, on the other hand, is known for its species that are processed into important products including vegetable oil, as well as phytochemicals with anti-inflammatory, antioxidant, hepatoprotective, and cognitive-enhancing properties [25, 26].

While the plants used in treating CNS/PNS disorders in Ghana varied greatly,* R. vomitoria* Afz. was frequently mentioned (17.5%) by the TMPs who had knowledge of natural products for treating these disorders. Herbal preparations of this plant are also used by TMPs in other African countries for the treatment of mental disorders [27] and have been shown to be relatively safe with LD_50_ of 17.5 g/kg [28]. Remarkably,* R. vomitoria* Afz. has been found to have activity on the nervous system, especially on locomotor behavior, anxiety, and psychosis [16, 18, 29]. Reserpine, which is one of the numerous alkaloids of this species, has been used in the management of schizophrenia, hypertension, and psychiatric disorders [30]. Beyond its CNS effect, extracts from the plant are reported to have anticancer (due to the alstonine and *β*-carboline alkaloid) [31], antipyretic, anti-inflammatory [32], and antidiabetic activities [33].

The natural products used by the TMPs in treating mental and neurological disorders fall into the following broad categories: analgesics, anxiolytics, antidepressants, antipsychotics, and anticonvulsants. Of these, those with analgesic (pain relieving), anxiolytic (anti-anxiety), and anticonvulsant (anti-epileptic) effects were the most commonly used, and this possibly reflects the common disorders treated by the TMPs. In particular, half of the identified TAMs were analgesics, possibly suggesting that the TMPs were most often presented with patients suffering from headache, migraine, or other associated conditions. Headache or cephalalgia is used to describe pain in the head and could be a symptom of a number of different conditions associated with the head and neck [1]. Although limited studies have been conducted to assess the epidemiology of headache and migraine in Ghana and Africa, headache is quite common among Africans and is often exacerbated by the hot climate in most African countries [34–36]. In assessing the profile of neurological disorders in an adult neurology clinic in Ghana, clinicians recorded a number of headache and migraine cases, although the frequency was found to be relatively low [37]. This low frequency was suggested to be due to the fact that primary headaches among Ghanaians are commonly reported to and managed by community pharmacists and primary healthcare physicians [37], although there are increasing reports indicating that several individuals with headache or migraine opt for traditional and herbal therapies [34, 35]. The analgesic species frequently used by the TMPs were* A. conyzoides *L. and* O. gratissimum *L. Also important are the anxiolytic (antianxiety) and anticonvulsant (antiepileptic) products that were often used by the TMPs, suggesting a potentially high prevalence of anxiety disorders and epilepsy and seizure disorders. Epilepsy, seizures, and anxiety disorders feature prominently among the mental and neurological conditions prevalent in Ghana [5, 37]. On the other hand,* L. camara *L. was the most frequently mentioned anxiolytic product, while* C. citratus *DC*., M. indica *L*., T. tetraptera *Schum Taub*.,* and* Persea Americana *Mill were among the most frequently studied anticonvulsants used by the TMPs.

Given that drugs for managing mental disorders are often in shortage in Ghanaian psychiatric hospitals [6, 38], it may be important that TAMs whose therapeutic relevance has been confirmed experimentally are considered for clinical usage. The long history of TAMs usage in African societies with seemingly minimal adverse effects [21, 39] support this perspective. While clinical integration of TAMs may be beneficial, at present, this should be approached with caution due to the inadequacy of studies exploring their efficacy and safety. Therefore, increasing TAMs-based research in Ghana would be a crucial step towards rigorous establishment of their safety, therapeutic, and adverse reaction profiles.

The natural products identified in this study are a valuable collection of resources that may provide leads for drug discovery and development. However, a potential criticism of the traditional approach being employed by the TMPs in relation to the pharmaceutical industry approach to drug discovery is that whole plant extracts may contain several bioactive components, making it difficult to attribute therapeutic benefits and mechanism(s) of action to particular compounds (Rasoanaivo et al., 2011). Moreover, some plant extract components may be negative modulators of active drug ingredients, with adverse implications for drug potential. A feasible means to refine, extend, and enhance the beneficial effects of the plant products identified in this study is to isolate, screen, and characterize bioactive compounds responsible for the positive disease-modifying effects reported. On the other hand, it is possible that components of the different plant extracts used in combination may produce positive interactions leading to complementarity in observed therapeutic effects that are more effective than single components administered at equal doses. In such a case, plant extracts whose benefits are observed when used as combinations by the traditional healers should be explored further to identify their possible synergistic activities. For example, the antimalarial drugs Quinimax® (a combination of quinone, cinchonine, and quinidine) and Malarone® (proguanil and avoquone) are produced and marketed as synergistic complementary drugs (Bunnag et al., 1989; Fivelman et al., 2004). Further drug discovery and development research should be conducted on the reported plant products to identify lead compounds whose* in vivo* therapeutic capacities would be revealed in preclinical and clinical studies. This would enable the industrial-scale production and marketing of successful drug candidates following drug authority approval. The high cost of the drug discovery and development process would, however, require strengthening academia-industry collaborative research and better provision of research funding and infrastructure [5, 40, 41].

## 5. Conclusion

The identified natural products used in Ghanaian communities are a potential source of a novel class of drugs for the management of mental and neurological disorders. Many of the plant species used have been investigated for their CNS-specific pharmacologic effects, with the majority having analgesic, anxiolytic, or anticonvulsant properties. However, the most prominent and often used plant,* R. vomitoria* Afz., has potent antipsychotic properties. The increased reliance and the claimed therapeutic value of the identified TAMs indicate that there is an urgent need for the preservation and extensive investigation of these products to establish their clinical effectiveness. Such studies may help in the isolation and purification of the bioactive compounds, confirm the safety and tolerability of these products, and enable the clinical integration of TAMs.

## Figures and Tables

**Table 1:** Sociodemographic characteristics of traditional medical practitioners who treat mental and neurological disorders in Ghana.

**Variable**	**Frequency**	**Percentage (**%**)**
**Sex**		
Male	37	56.1
Female	29	43.9

**Age (years)**		
20-29	1	1.5
30-39	8	12.1
40-49	12	18.2
50-59	27	40.9
60 and above	18	27.3

**Marital status**		
Single	15	22.7
Married	43	65.2
Divorced	1	1.5
Widowed	7	10.6

**Highest educational level**		
No Education	5	7.8
Primary	34	53.1
Secondary	18	28.1
Tertiary	7	10.9

**Table 2:** Sources of knowledge and duration of practice for traditional medicine practitioners who treat mental and neurological disorders in Ghana.

**Variable**	**Frequency**	**Percentage (**%**)**
**Source of the knowledge of herbal medical practice**		
Inheritance (knowledge passed on from others)	38	63.3
Divine/spiritual	14	23.3
Formal training	8	13.3

**Years of herbal medical practice **		
1-5	17	27.0
6-10	10	15.9
11-15	13	20.6
16-20	23	36.5

**Table 3:** Treatment of mental and neurological disorders by traditional medical practitioners in Ghana.

**Variable**	**Frequency (n)**	**Percentage (**%**)**
**Knowledge of herbs for treating mental and neurological disorders**		
No	26	39.4
Yes	40	60.6

**Total number of patients treated throughout herbal medical practice **		
0	42	63.6
1-5	13	19.7
6-10	5	7.6
11-15	3	4.5
16-20	2	3.0
Above 20	1	1.5

**Treatment options used **		
Not applicable	42	63.6
Divine/spiritual only	1	1.5
Herbs only	16	24.3
Herbs and divine/spiritual	7	10.6

**Recovery status of patients treated **		
Not applicable/no recovery	42	63.6
Partial recovery	3	4.5
Total recovery	21	31.8

**Table 4:** Local names, plant parts, and methods of preparation of traditional African medicines used in treating nervous system and mental disorders in Ghana.

**Species**	**Family **	**Growth forms **	**Local names in different languages**	**Voucher specimen **	**Frequency of mention**	**Plant part used**	**Method of preparation **
*Ageratum conyzoides*	Asteraceae	Herb	**Ewe**: mima, Nyigbe; **Fante**: Ahaban Kankan, Efumomoe; **Twi**: Gu-ekuro, Adwoa-kura, Guakuo, Gu-ekura; **Nzema**: Ebuakulo; **Ga-Dangme**: Ntumumu	PA01/UGSOP/GH17	3	Leaves	The fresh leaves are macerated and the liquid obtained is instilled into the nostrils; the fresh leaves can also be boiled, sieved and drank as required.
*Allium sativum*	Liliaceae	Herb	**Twi**: Gyene Kankan; **Ga Adangme**: Aya; **Hausa**: Tafarmuwa	PA02/UGSOP/GH17	2	Whole plant	-
*Alstonia boonei*	Apocynaceae	Tree	**Twi: **Nyame-dua, Nyamedua, Onyamedua, Osen Nuru; **Ewe: **Siaketreke **Fante: **Nyena,Sinuro,Nyamedua; **Nzema: **Bakunin	PA03/UGSOP/GH17	2	Bark	The leaves are boiled and drank as required
*Azadiratcha indica*	Meliaceae	Tree	**Fante: **Nim, Aboode,Abodua; Ewe: Liliti; **Ga-Dangme**: Kintso, **Asante**: Gyedua; **Twi: **Nimsi, Dua gyane	PA04/UGSOP/GH17	1	Leaves; Roots	The boiled leaves/roots are drank as required
*Bertholletia excels*	Lecythidaceae		Brazil nut	PA05/UGSOP/GH17	1	Nut; Leaves	The leaves/nuts are boiled and sieved extract is drank as required
*Bidens pilosa.*	Asteraceae	Herb	**Twi: **Dwirentwi,Gyinantwi; **Ewe: **Dzanikpikpi;	PA06/UGSOP/GH17	1	Leaves	The fresh leaves are macerated and the liquid obtained is instilled into the nose
*Blighia unijucata*	Sapindaceae	Tree	**Asante**: Akye, Akan, Akyibiri, **Twi: **Akyebiri, **Fante: **Etedua	PA07/UGSOP/GH17	3	Bark; Roots	The dried barks/roots are boiled and drank as required, the extract can also be smeared on the body
*Cassia occidentalis.*	Caesalpiniaceae		Nkwadowa b*ɔ*deɛ	PA08/UGSOP/GH17	3	Leaves	The leaves are boiled and drank as required
*Cinchona pubescens*	Rubiaceae	Shrub		PA09/UGSOP/GH17	1		
*Citrus aurantifolia*	Rutaceae		**Twi: **Ankaadwea, Akenkaa Ankaatwaree; **Fante**: Ankama, **Ewe**: Mumoe; **Asante**: Ankaatwaree; **Dagbani**: Nyamsa, Lim buri; **Ga-Adangme**: Abonua; **Hausa: **Olomankilisi; **Nzema: **Domunli; **Mole: **Leemu; **Ga: **Kpete	PA10/UGSOP/GH17	1	Peel; Juice	The peels are squeezed directly on the forehead and into the nose
*Cocos nucifera*	Palmae	Tree	**Twi: **kube; **Ewe**: Agone	PA11/UGSOP/GH17	1	Juice	Drinking the fresh coconut juice at will
*Corchorus olitorius*	Tiliaceae	Herbaceous	**Ewe**: Ademe,Singui; **Fante: **Oturo; **Twi**: Otoro	PA12/UGSOP/GH17	1	Jute mallow, Leaves	Hot infusion is made from the leaves and drank as required
*Cymbopogon citratus*	Graminae	Herb	**Ewe**: Tigbe; **Fante: **Ti ahaban, **Ga-Dangme:**Ti-ba	PA13/UGSOP/GH17	1	Leaves; Oil	A decoction is made from the either the fresh/dried leaves and the oily content applied as a massage
*Eucalyptus globulus *	Myrtaceae		Eucalyptus	PA14/UGSOP/GH17	1	Oil	Cold infusion is made and the oily content obtained is rubbed on the body
*Khaya senegalensis*	Meliaceae	Tree	**Hausa: **Madwachi, Madachi; **Ewe: **Logo; **Fante: **Okum; **GaAdangme: **Kuga; **Twi**: Kuntunkuri; **Mole**: Kuka; **Brong**: Korobaa; **Nzema: **Anane	PA15/UGSOP/GH17	4	Bark	The leaves are boiled and drank as required
*Lantana camara *	Verbenaceae	Shrub	**Akan**,Ananse dokono	PA16/UGSOP/GH17	3	Leaves; Stem	The leaves/stem are boiled and the liquid obtained, drank as required
*Mangifera indica*	Anacardiaceae	Tree	**Ewe/Asante/Twi/ Fante** Mango, Amango,**Ga: **Mango	PA17/UGSOP/GH17	1	Bark	A decoction is made from the dried bark and drank as required
*Momordica charantia*	Cucurbitaceae	Herbaceous	**Twi**: Nyannya, Nyina, Nyinya; E**we**: Kakle; **Dangme**: Nyanyla, Nyanyra; **Ga**: Nyanyra; **Nzema: **Nyanya	PA18/UGSOP/GH17	1		
*Musa paradisiaca*	Musaceae	Herbaceous	**Twi**: Brode; **Nzema**: Banna **Ga: **Amadaa	PA19/UGSOP/GH17	1	Leaves	The leaves are boiled and drank as required
*Nephrolepis cordifolia *	Nephrolepidaceae		**Twi:** Mmɛn	PA20/UGSOP/GH17	1	Leaves	The leaves are macerated and the liquid instilled nasally or inhaled. The leaves can be boiled and the extracted liquid used as a bathing liquid.
*Occimum gratissimum*	Lamiaceae	Shrub	**Ewe: **Babusui, Dzeveti; **Ga: **Sulu; **Twi: **Onunum, Nunum; **Asante: **Nunum; **Ga-Dangme: **Sulu; **Hausa: **Dardoyatagidi; **Nzema: **Amaloko; **Wassa: **Aprim; **Fante: **Onunum	PA21/UGSOP/GH17	3	Leaves	The leaves are boiled and drank as required
*Passiflora edulis*	Passifloraceae		Passion fruit tree	PA22/UGSOP/GH17	2	Leaves; Flowers; Fruit; Leaves	Boiling; grinding
*Persea americana*	Lauraceae	Tree	**Dangme: **Paya; **Twi: **Pee; **Akan: **Paya, Pae;	PA23/UGSOP/GH17	1	Fresh and dried leaves	A decoction is made from the either the fresh/dried leaves and drank as required
			**Fante**: Pae;				
*Phyllantus nuriri*	Euphobiaceae	Herbaceous	**Twi: **Awommaguwakyi; **Ewe**: Lane; **Krobo: **Ofobiokpai, Ofobi; **Ga: **Omatsoatsi;	PA24/UGSOP/GH17	1		
*Rauwolfia vomitoria.*	Apocynaceae	Shrub	**Twi: **Kakapenpen; **Ewe: **Dodemakpowoe; **Fante: **Kakapenpen; **Ga-Dangme: **Apototso; **Hausa: **Wada, **Nzema: **Bakapembene; **Wassa: **Aneene	PA25/UGSOP/GH17	7	Roots	The roots are boiled and the extract obtained are instilled into the nose
*Rubus fruticosus*	Rosaceae		Bramble	PA26/UGSOP/GH17	1	Berries, leaves and flowers	Blend dry leaves and mix with honey
*Scoparia dulcis *	Scrophulariaceae			PA27/UGSOP/GH17	1	-	-
*Sida acuta *	Malvaceae	Branchlets	**Ewe: **Afideme; **Ga: **Shwoboto; **Twi: **Obraneatuto	PA28/UGSOP/GH17	1	Leaves	The leaves are boiled and drank as required
*Tapinanthus globiferrus*	Loranthaceae	Parasitic Tree	**Twi: **nkranpan **Mole: **Welebe	PA29/UGSOP/GH17	2	Leaves; stem	The leaves/stem are boiled and the liquid obtained, drank as required
*Terminalia catapa*	Combretaceae		abr*ɔ*fo nkateɛ	PA30/UGSOP/GH17	1	Yellowed leaves	The leaves are boiled and the liquid drank as required
*Tetrapleura tetraptera*	Fabaceae	Tree	**Twi**: Prɛkesɛ, **Zate**: Zamturi; **Anyi: **Aprekese, Kyeke, **Fante: **Esem, **Ewe: **Prekese	PA31/UGSOP/GH17	1	Seed	The seeds are ground and the liquid extract drank as required
*Vernonia amygdalina*	Asteraceae	Shrub	**Ga:**Tatso, Akpa, **Dagbani: **Biebingira, **Ewe: **Gbo, Gboti, **Asante: **Mbonasere, Mponasere; **Nzema: **Ayeanwole, **Ga-Dangme: **Tatsho	PA32/UGSOP/GH17	1	Leaves	A decoction is made from the either the fresh/dried leaves and drank as required

**Table 5:** Plant species used for the treatment of mental and nervous system disorders in Ghana and scientific validation of their ethnopharmacological activities.

**Botanical name**	**Family **	**CNS uses**
*Ageratum conyzoides *Linn.,	Asteraceae	Analgesia [15, 42, 43]
*Allium sativum *Linn.	Liliaceae	Motor coordination[44]; Analgesia [45]
*Alstonia boonei *De Wild	Apocynaceae	Analgesia [24]

*Azadirachta indica *A. Juss	Meliaceae	Analgesia [25, 46]; Anxiolytic [47]; Alzheimer's disease [26]

*Bertholletia excelsa *H&B	Lecythidaceae	None

*Bidens pilosa *Linn.	Asteraceae	Analgesia [48]
*Blighia unijugata *Bak	Sapindaceae	None

*Cassia occidentalis *Linn.	Caesalpiniaceae	None
*Cinchona pubescens* Vahl.	Rubiaceae	None
*Citrus aurantifolia *Swingle	Rutaceae	None

*Cocos nucifera *Linn.	Palmae	Analgesia [49–51]

*Corchorus olitorius *Linn.	Tiliaceae	Anticonvulsant [52]

*Cymbopogon citratus *DC.	Graminae	Anxiolytic [53–56]; Sedative [53]; Anticonvulsant [53, 54, 57]; Analgesia [58]

*Eucalyptus globulus *Labill.	Myrtaceae	None
*Khaya senegalensis *(Desr.) A. Juss.	Meliaceae	None
*Lantana camara *Linn.	Verbenaceae	Anxiolytic [59, 60]
*Mangifera indica *Linn. F.T.A	Anacardiaceae	Analgesia [61, 62];
		Cognitive performance [63]
		[64]; Neuroprotection, anticonvulsant [65]

*Momordica charantia *Linn.	Cucurbitaceae	Analgesia [66–68]; Antidepressant Anxiolytic [69]

*Musa paradisiaca *Walker et Sillans	Musaceae	None

*Nephrolepis cordifolia *Linn Presl	Nephrolepidaceae	None

*Ocimum gratissimum *Linn.	Lamiaceae	Analgesia, antidepressant [70–74]; and anxiolytic [75]

*Passiflora edulis *Sims	Passifloraceae	Anxiolytic [76–81] and sedative [78, 81, 82]

*Persea Americana *Mill F.W.T.A	Lauraceae	Analgesia and anticonvulsant [83, 84]

*Phyllanthus niruri *Schum.et Thnn.	Euphobiaceae	Analgesia [85, 86]
*Rauwolfia vomitoria *Afz.	Apocynaceae	Antipsychotic [16, 18, 29]
*Rubus fruticosus *Linn.	Rosaceae	None
*Scoparia dulcis *Linn.	Scrophulariaceae	Analgesia [87, 88]
*Sida acuta *Burn F.	Malvaceae	Analgesia and antidepressant [89, 90]

*Tapinanthus globiferus *A. Rich.	Loranthaceae	None
*Terminalia catappa *Linn.	Combretaceae	None
*Tetrapleura tetraptera *Schum Taub.	Fabaceae	Anticonvulsant [91, 92], Analgesia [92]
*Vernonia amygdalina *Del. Cent. Pl. Afr.	Asteraceae	Analgesia [22]

## Data Availability

The data used to support the findings of this study are available from the corresponding author upon request.

## Abbreviations

CNS: Central nervous system
GHAFTRAM: Ghana Federation of Traditional Medical Practitioners' Association
GPs: General practitioners
TAMs: Traditional African medicines
TMPs: Traditional medicine practitioners.
